# Integrating transformers and many-objective optimization for drug design

**DOI:** 10.1186/s12859-024-05822-6

**Published:** 2024-06-08

**Authors:** Nicholas Aksamit, Jinqiang Hou, Yifeng Li, Beatrice Ombuki-Berman

**Affiliations:** 1 Department of Computer Science, Brock University, 1812 Sir Isaac Brock Way, St. Catharines, ON L2S 3A1 Canada; 2 Department of Biological Sciences, Brock University, 1812 Sir Isaac Brock Way, St. Catharines, ON L2S 3A1 Canada; 3 Department of Chemistry, Lakehead University, 955 Oliver Road, Thunder Bay, ON P7B 5E1 Canada; 4 Thunder Bay Regional Health Research Institute, 980 Oliver Road, Thunder Bay, ON P7B 6V4 Canada

**Keywords:** Drug design, Molecular generation, Transformers, Many-objective optimization, Evolutionary algorithm, Particle swarm optimization, ADMET, LPA1

## Abstract

**Background:**

Drug design is a challenging and important task that requires the generation of novel and effective molecules that can bind to specific protein targets. Artificial intelligence algorithms have recently showed promising potential to expedite the drug design process. However, existing methods adopt multi-objective approaches which limits the number of objectives.

**Results:**

In this paper, we expand this thread of research from the *many*-objective perspective, by proposing a novel framework that integrates a latent Transformer-based model for molecular generation, with a drug design system that incorporates absorption, distribution, metabolism, excretion, and toxicity prediction, molecular docking, and *many*-objective metaheuristics. We compared the performance of two latent Transformer models (ReLSO and FragNet) on a molecular generation task and show that ReLSO outperforms FragNet in terms of reconstruction and latent space organization. We then explored six different *many*-objective metaheuristics based on evolutionary algorithms and particle swarm optimization on a drug design task involving potential drug candidates to human lysophosphatidic acid receptor 1, a cancer-related protein target.

**Conclusion:**

We show that multi-objective evolutionary algorithm based on dominance and decomposition performs the best in terms of finding molecules that satisfy many objectives, such as high binding affinity and low toxicity, and high drug-likeness. Our framework demonstrates the potential of combining Transformers and *many*-objective computational intelligence for drug design.

## Introduction

Humans are constantly under the threat of pain and disease, and a method for treatment of both is the administration of drugs. However, the drug development process is lengthy and monetarily expensive, with estimated development period of 10–15 years and cost between $90 million to $2.6 billion [1]. Furthermore, since the later stages of drug development rely on the success of earlier ones, failures may result in a repetition of earlier stages, thereby prolonging the duration of development and increasing costs. Artificial Intelligence (AI), and particularly deep learning [2], have provided promising approaches that address some limitations of the current drug development pipeline, and provided an efficient method of traversing through the large chemical space of estimated $$10^{60}$$ molecules [3]. Contemporary deep learning architectures and strategies such as Transformers, geometric learning, and reinforcement learning, have all been applied to drug-related tasks such as molecular property prediction, molecular generation, and drug design [4–6].

However, most of the existing works focus on effective modelling of molecular representation and search in the chemical space, but did not fully consider the requirements and factors of failures in the process of drug discovery and development. As reviewed in [7], most of the existing works in drug design adopt a multi-objective approach, which optimizes two or three objectives at a time, or scalarize the objectives by use of an aggregation function. However, this fails to capture the full outlook of the drug design problem, which involves many conflicting and interrelated objectives. In [8, 9], the authors introduce a system named molecule swarm optimization (MSO) for drug design using a latent neural translation model for molecular generation and molecular optimization with particle swarm optimization (PSO) and a scalarized objective via a weighted linear combination of multiple objectives. It has been shown in the work of deep evolutionary learning (DEL) [10], the Pareto treatment of multiple objectives, including quantitative estimate of drug-likeness (QED), log octanol-water partition coefficient (logP), and synthetic accessibility score (SAS), outperforms the scalarization treatment in molecular generation. From our perspective, drug design can be naturally modelled as a many-objective optimization problem, because a good drug candidate needs to satisfy many physio-chemistry properties to make sure that it is drug-like, less toxic, and more effective. Consistent with [7], many-objective optimization in our research deals with more than three objectives.

To enhance the earlier stages of drug discovery and development, this work reformulates the drug design problem as molecular generation and *many*-objective optimization tasks, via combining of Transformer (the state-of-the-art sequence modelling and generation technique) and many-objective metaheuristics based on evolutionary algorithms and particle swarm optimization (for effective metaheuristic search within the vast chemical space). Our framework improves upon existing works by utilizing a Transformer-based latent model for molecular generation, ADMET (absorption, distribution, metabolism, excretion, and toxicity) objectives, molecular docking, and many-objective metaheuristic algorithms. As popularized in literature, Transformers [11] have surpassed the performance of recurrent neural networks (RNNs) for many natural language processing tasks. However, the vanilla Transformer has no explicit latent space, such as those found in RNN autoencoders, as used in [8]. There exist works in literature that construct a Transformer-based autoencoder, such as FragNet (a contrastive learning-based Transformer model) [12] and ReLSO (Regularized Latent Space Optimization) [13]. However, these architectures employ differing approaches, such as contrastive learning in FragNet, and property prediction, along with three regularization penalty terms in ReLSO. Therefore, to understand the performance of latent Transformer-based models in molecular generation and employ the superior for molecular optimization, we performed a fair comparative analysis between these two models.

Furthermore, to the best of the authors’ knowledge, the performance of many-objective optimization approaches in the drug design domain had been unknown before this work. Thus, we performed a comparative analysis among many-objective metaheuristics applied to a drug design problem. To address the limitations of single- or multi-objective approaches, this study employs a Pareto-based *many*-objective optimization approach, which handles more than three objectives and generates a set of high-quality drug candidates that represent trade-offs among the objectives. As well, we include binding affinity, and ADMET properties as objectives, noting that 40–50% of drug candidates fail due to poor efficacy and 10–15% of candidates fail from inadequate drug-like properties [14]. During the writing of this paper, we became aware of a recent work that applies a metaheuritic algorithm within the latent space of a Transformer-based autoencoder model for drug design [15], however the authors use a multi-objective, rather than a many-objective, problem, and do not include ADMET objectives or molecular docking.

We outline the contributions of this paper as follows:

1. We necessarily evaluated the performance between two latent Transformer models, ReLSO and FragNet, for molecular generation.
2. To the best of our knowledge, this is the first study to comprehensively evaluate the performance of *many*-objective computational intelligence algorithms for drug design problem.
3. We propose a system which integrates a predictive Transformer and a generative latent Transformer with many-objective computational intelligence algorithms and molecular docking.
4. To the best of our knowledge, we are the first to incorporate *many*-objective computational intelligence algorithms in the latent space of a generative Transformer, while also using ADMET-related objectives and binding affinity as objectives.

The rest of this paper is organized as follows: "Related work" section describe works closely related to the experiments performed in this study. Afterwards, background information on the implemented methods are discussed in "Methods" section. Following is "Experiments" section, where we outline the evaluation methods used for assessing our experiments, and provide both results and discussion. Lastly, in "Conclusion" section, we explain limitations to our studies and list future avenues of work.

## Related work

**Table 1:** SMILES and SELFIES representations of carbinoxamine

Name	Example
SMILES	CN(C)CCOC(C1=CC=C(C=C1)Cl)C2=CC=CC=N2
SELFIES	[C][N][Branch1][C][C][C][C][O][C][Branch1][N][C][=C][C][=C][Branch1]
	[Branch1][Branch1][C][=C][Ring1][=Branch1][Cl][C]
	[=C][C][=C][C][=N][Ring1][=Branch1]

The Simplified Molecular-Input Line-Entry System (SMILES) [16] and SELF-Referencing Embedded Strings (SELFIES) [17] are two prevalent line notations for chemical language modelling. SMILES, which is more widely used, has a non-unique but unambiguous representation of molecules, implying that a single molecule can have multiple corresponding strings, but each string can only denote one molecule. SELFIES is derived from applying context-free grammar rules to encode a SMILES string, and it ensures the validity of the generated molecule. This is an advantage over SMILES notation in molecular generation, which often produces invalid molecules due to its rigid syntactic rules. A valid SMILES molecule can be obtained from decoding any sequence of SELFIES symbols, and a unique SELFIES string can be generated from encoding any SMILES molecule. In this study, we use SMILES for ADMET prediction to match our selected base model implementation, and SELFIES for molecular generation to guarantee validity of generated molecules. Table 1 shows an example of a SELFIES and SMILES string for carbinoxamine.

Transformers have been widely applied for molecular representation learning. However, many works either do not construct a readily available latent space for optimization, as in Uni-Mol [18], or construct a latent space, but do not implement a decoder for generating a molecule, as in KPGT [19], MM-Deacon [20], and GeoT [21]. We require a latent space, along with a decoder, to construct a decision space for optimization, along with the ability to generate a molecule from the vectorized latent representation. Some models that fit this criteria include SMILES Transformer [22], FragNet [12], and MolMIM [15]. For our experimentation, we employ the FragNet architecture over SMILES Transformer, as it uses learnable compression methods and contrastive learning for latent space regularization. Additionally, we make use of ReLSO (regularized latent space optimization) [13], an architecture constructed for protein sequence generation and optimization, which we deem a problem similar to the experiments performed in this study.

In addition to molecular representation learning, ADMET prediction, and molecular generation, we developed a drug design system and executed a comparative analysis with various many-objective metaheuristics. Metaheuristic optimization has been widely used for drug design, either by building molecules fragment-by-fragment or atom-by-atom [23–27]. Furthermore, with recent advances, metaheuristic algorithms have been combined with deep learning models to explore novel chemical spaces. Some examples of studies that incorporate a latent space are MSO [9] and the DEL framework [10, 28, 29]. A distinctive feature of the DEL framework is that it actively trains the generative model with the molecules generated by the metaheuristic algorithm, which regularizes the latent space to facilitate the optimization process. We note that these studies use a multi-objective approach and do not employ a Transformer-based backbone. In the case of DEL, these works do not incorporate ADMET properties as objectives, while in MSO, the scalarization approach is used. We take a Pareto-based many-objective approach, recognizing the importance of trade-off solutions in conflicting objective functions, and incorporate ADMET properties and molecular docking in our drug design system.

## Methods

**Fig. 1:**
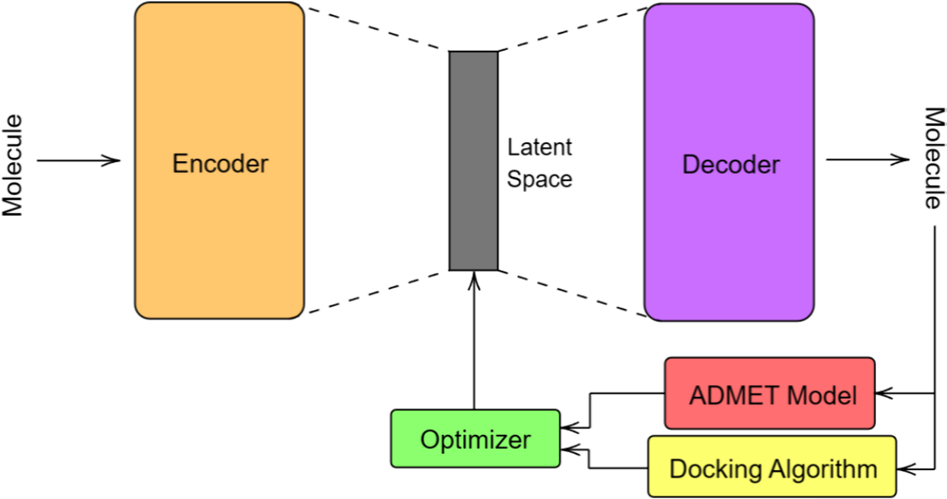
Our many-objective drug design system

In this section we describe the methods used in our proposed drug design framework, which is illustrated in Fig. 1. This includes the Transformer-based models for molecular generation in "Contrastive transformers for molecular generation" section, and many-objective metaheuristics, along with remaining modules such as ADMET prediction and docking algorithm, in "Many-objective drug design from the latent space" section.

### Contrastive transformers for molecular generation

Contrastive learning is a self-supervised technique used to learn meaningful representations of data by comparing latent vectors against positive and negative samples [30]. Since SMILES is a non-unique representation, a given molecule may be enumerated by many SMILES strings, which facilitates the generation of positive and negative pairs. Equations (1) and (2) illustrate the normalized temperature-scaled cross entropy (NT-Xent) loss, where positive latent samples $z_i$ and $z_j$ have their cosine similarity maximized by contrasting with remaining samples in a mini-batch of length *N*, which is repeated for all pairs of positive samples, with a temperature parameter $\tau$. We note that in Eq. (1) and (2), 2*N* is used as only two positive samples are considered for each SMILES string.

1 $$\begin{aligned} l(i,j)= & {} -\textrm{log}\frac{\textrm{exp}(\textrm{sim}(z_i,z_j) / \tau )}{\sum _{k=1}^{2N} 1_{[k \ne i]} \textrm{ exp}(\textrm{sim}(z_i,z_k) / \tau )}. \end{aligned}$$

2 $$\begin{aligned} L= & {} \frac{1}{2N} \sum _{k=1}^N [l(2k-1, 2k) + l(2k, 2k-1)]. \end{aligned}$$

**Fig. 2:**
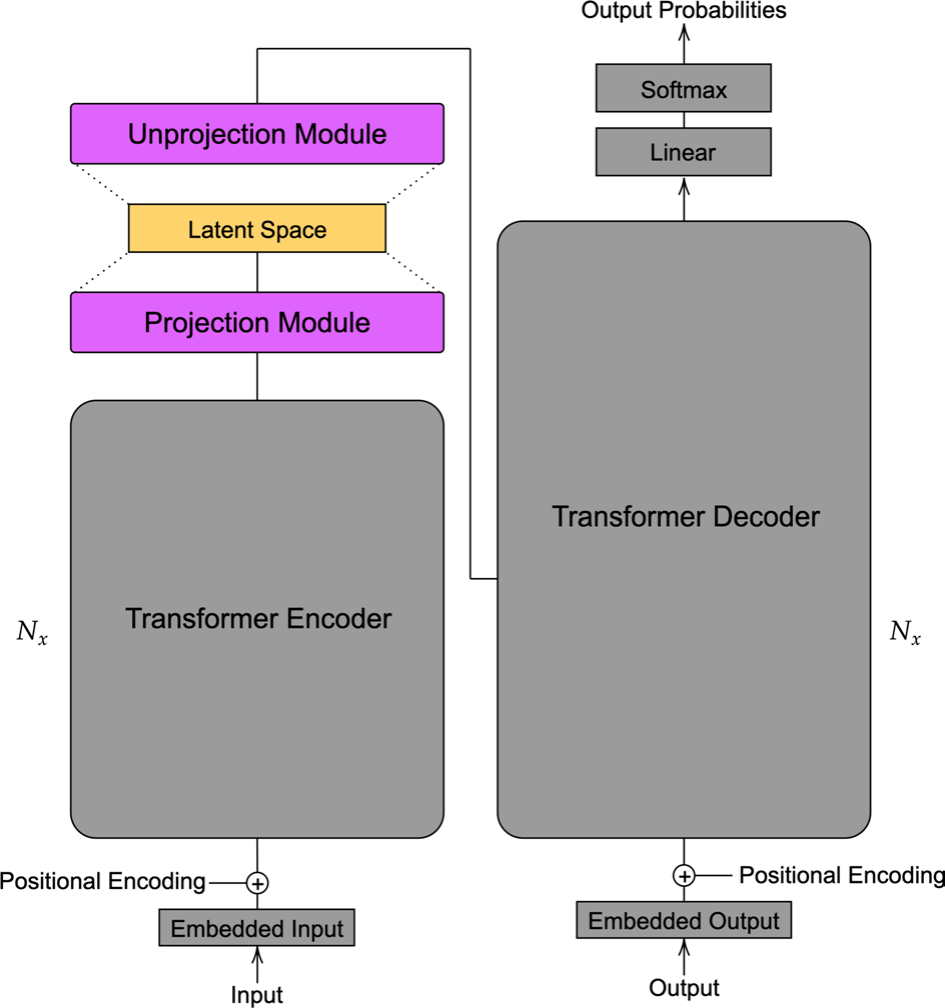
Architecture of FragNet

**Fig. 3:**
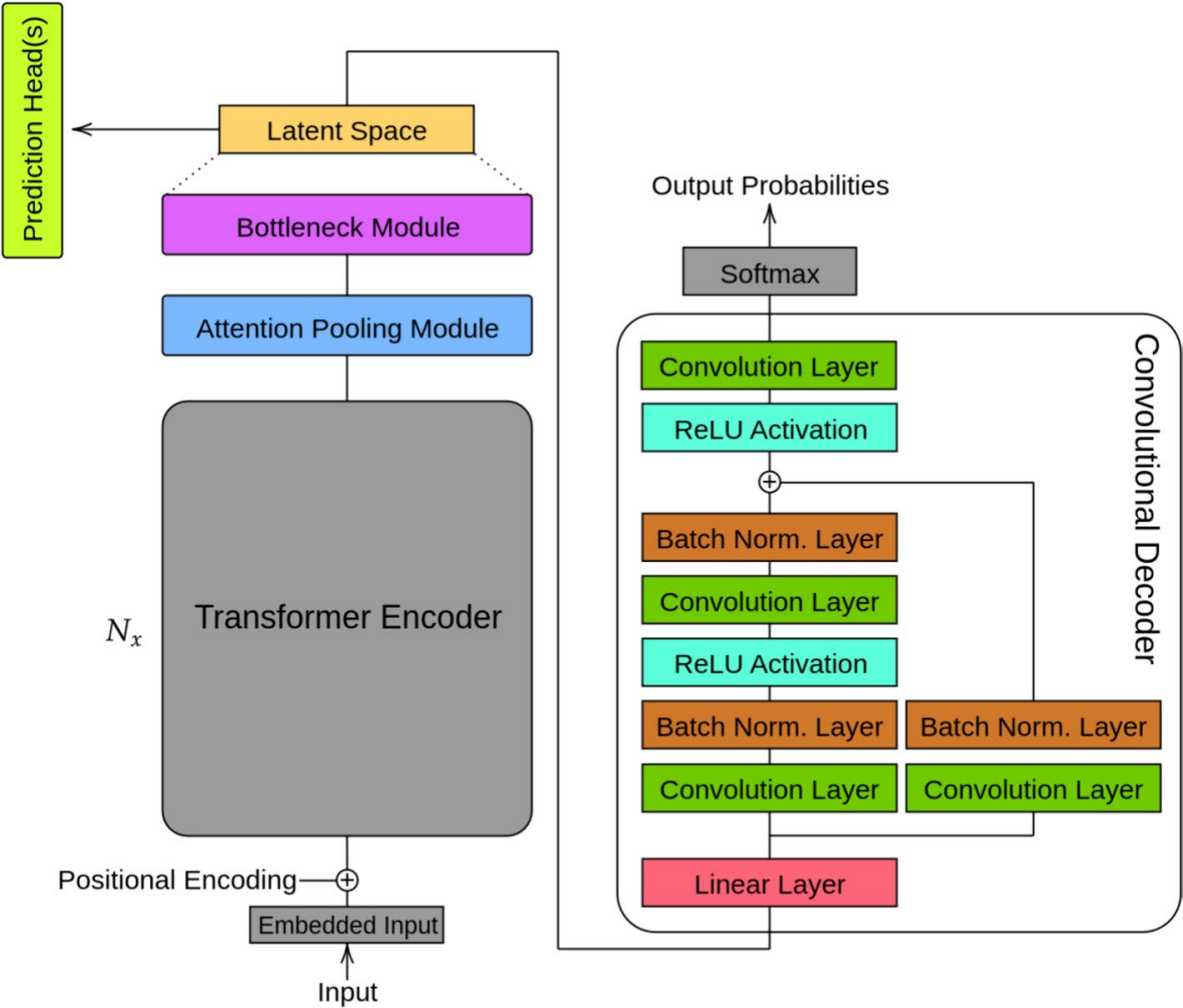
Architecture of ReLSO

Using contrastive learning, we apply two latent Transformers for molecular generation, FragNet [12] and ReLSO [13], and determine the best model for latent molecular representation. Figures 2 and 3 illustrate the architectures of FragNet and ReLSO. After our experiments, the leading latent Transformer model was applied on a many-objective drug design task. FragNet adapts the standard Transformer architecture by inserting projection and unprojection modules in-between the encoder and decoder. These modules allow the model to transform the encoder output into a latent representation vector, and vice versa. Structurally, the projection and unprojection modules consist of four linear layers with the ReLU activation function. We further *alter* the FragNet model to include prediction heads from the latent space and a latent vector $l_2$ norm penalty, as seen in the ReLSO work. This allows for fair comparison between the two architectures, and also regularizes the latent space by molecular properties, which is important for downstream optimization.

ReLSO was originally proposed for protein sequence generation and optimization, and consists of a Transformer encoder, projection module, convolutional decoder, with prediction heads and three penalty terms in the loss function for regularization [13]. In this study, we *repurpose* ReLSO for small molecule modelling by modifying its latent space regularization, and also constructing both a contrastive learning and non-contrastive learning variant to investigate changes in performance. Firstly, we replace the interpolative sampling and negative sampling penalty with contrastive learning. We do this as contrastive learning regularizes the latent space by distancing molecules that are different while keeping similar molecules closer together, similar to interpolative sampling. We do not employ negative sampling penalty, as in our drug design system we calculate the objective values such as ADMET properties external to the molecular generation model. As a result, the latent vector $l_2$ norm penalty remains from the original ReLSO study. Similar to our FragNet implementation, we perform a joint-training task for sequence modelling, property prediction, contrastive learning, and with the latent $l_2$ norm penalty.

### Many-objective drug design from the latent space

To explore the potential of *many*-objective metaheuristic algorithms for drug design, we performed a comparative study by applying six well-known and robust many-objective metaheuristic algorithms for a drug design task. As part of the evolutionary multi-objective optimization platform (PlatEMO) [31], we employed many-objective metaheuristics that cover four of the five many-objective optimization approaches discussed in [32], and leveraged the latent space of the best molecular generation model from our experiments. We list our selected metaheuritics with a brief description, as follows:

- *Grid-based Evolutionary Algorithm (GrEA)* [33]: An evolutionary algorithm that partitions the objective space into a grid structure and maintains a representative solution for each grid cell.
- *Hypervolume Estimation (HypE)* [34]: An evolutionary algorithm that utilizes a Monte Carlo estimation of the hypervolume indicator to select and rank solutions.
- *Knee Point-driven Evolutionary Algorithm (KnEA)* [35]: An evolutionary algorithm that incorporates knee-point information into the mating and environmental selection mechanisms to guide the search towards the most preferred solutions.
- Multi-objective Evolutionary Algorithm Based on Dominance and Decomposition (MOEA/DD) [36]: A decomposition-based evolutionary algorithm that employs weight vectors, Pareto-dominance, and density measures to order solutions.
- *Adaptive Non-Dominated Sorting Genetic Algorithm III (A-NSGA-III)* [37]: An evolutionary algorithm that adapts the number and location of reference points according to the distribution and convergence of solutions, and applies a niche preservation strategy to maintain diversity.
- *Novel Multi-objective Particle Swarm Optimizer (NMPSO)* [38]: A particle swarm optimization algorithm that adopts a balanceable fitness estimation method to balance the convergence and diversity of the swarm, and applies a mutation operator to escape from local optima.

For each of these algorithms, an initial population is generated by randomly sampling the molecules from the dataset used for constructing the molecular generation models, and transforming them into latent vectors through the encoder.

As illustrated in Fig. 1, after an optimization step is performed, the corresponding vector is transformed into a molecule through the decoder, where objective values are then obtained by applying the molecule to an ADMET model and docking algorithm. After decoding the latent vector, we also encode the corresponding molecule to repair its positioning in the latent space, as is performed in [9]. Thus, our drug design framework heavily relies on a Transformer-based autoencoder, with a supporting ADMET model and docking algorithm for objective prediction.

During the optimization process we use binding affinity, synthetic accessibility score (SAS), bioavailability, solubility, acute toxicity LD50, and ClinTox toxicity prediction as objectives. SAS is a scaled measure from 1 to 10, with lower values corresponding to ease of synthesizability. We use the method by Ertl and Schuffenhauer [39] to obtain SAS values, as implemented in RDKit [40]. Binding affinity is predicted using a GPU-accelerated QuickVina2 implementation, with lower values signalling higher ligand-protein affinity [41]. For the remaining objectives, we trained and used multi-task learning BERT (MTL-BERT) which is a state-of-the-art ADMET prediction model [42] based on Bidirectional Encoder Representations from Transformers (BERT) [43]. In this case, MTL-BERT was pretrained using SMILES strings of 4 million molecules (from ZINC-250K [44], ChEMBL [45], and MOSES [46]) and then fine-tuned by adding 29 heads corresponding to 29 ADMET tasks using data from [47]. The selected ADMET objectives satisfied our criterion of having their respective area under the receiver operating characteristic curve (AUC) or coefficient of determination ($R^2$) performance measures higher than a value of 0.8, where bioavailability and solubility are to be maximized, with minimization for all remaining ADMET objectives.

## Experiments

In "Data and hyperparameters" section we outline our experimental setup, including parameters for training the molecular generation models and metaheuristic parameters for optimization. Moreover, we specify the data used for training our molecular generation models and ADMET prediction model. Afterwards, in subsequent "Comparing transformers for latent space generation" and "Comparing computational intelligence methods for molecular optimization" sections, we indicate our methods of evaluation, and provide corresponding results to our experiments. We conclude by presenting a case study for our drug design system in "Case study" section.

### Data and hyperparameters

**Table 2:** QuickVina2-GPU docking parameters on LPA1 protein

Parameter	Value
Center (x, y, z)	(14.444, 5.250, − 18.278)
Size (x, y, z)	(20, 20, 20)
Invalid docking value	1000000

**Table 3:** Molecular generation model hyperparameters

Name	Value
Learning rate (FragNet/ReLSO)	1e−5/2e−5
Batch size	64
Embedding dimension	256
Latent dimension	512
Transformer layers	6
Self-attention heads	8
Feedforward dimension	1024
Dropout	0.2
Recon loss weight	1
Auxiliary weights	[0.25, 0.25, 0.25]
Latent L2 penalty weight	0.1
Contrast weight	1
Temperature	0.1
Random seeds	[42, 182, 625, 511, 310]

**Table 4:** Metaheuristic algorithm parameters

Algorithm	Parameters
NMPSO	$\omega \in [0.1, 0.5]$, $c_1, c_2, c_3 \in [1.5, 2.5]$
KnEA	$T = 0.5$
GrEA	$div = 8$
HypE	$nSample = 10000$
MOEA/DD	$\delta = 0.9$, $T = 200$, $\theta = 5$
Global parameter	Value(s)
Population size	2000
Function evaluations	25000
Random seeds	[42, 182, 625, 511, 310]

Tables 2 and 3 illustrate the parameters used for docking via QuickVina 2-GPU [41] and molecular generation models. Molecular docking was performed on lysophosphatidic acid receptor 1 (LPA1), a protein that is implicated in a diverse array of cellular activities that promote cancer cell migration, and invasion [48–50]. The PDB file of LPA1 was downloaded from the AlphaFold Protein Structure Database (AlphaFold Entry: Q92633), and then processed using AutodockTools [51] to obtain the PDBQT file as one of the input files for QuickVina 2-GPU docking. LPA1 is one of six GPCRs in the LPA receptor family (LPA1-6), and is activated by the bioactive phospholipid, lysophosphatidic acid (LPA). LPA acts like a growth factor that stimulates a wide range of cellular responses, such as calcium mobilization, cell proliferation, cell migration, and chemotaxis [52, 53]. Activation of LPA1 by LPA is implicated in a diverse array of cellular activities that regulate cell proliferation, migration, and invasion [48, 53]. It has been reported that the mRNA expression of LPA1 is elevated in advanced stages of breast cancer compared with early stage [54]. In several breast cancer cell lines including Triple Negative Breast Cancer (TNBC), the expression of LPA1 is significantly higher compared with non-tumorigenic cell line and activation of LPA1 by LPA stimulated cell migration and invasion in breast cancer cell lines in vitro, while LPA antagonists inhibited the effects of LPA-induced proliferation and migration [55, 56]. In mouse models of breast cancer, the overexpression of LPA1 was found to enhance tumor growth and promote metastasis to the bone [57]. Conversely, silencing or pharmacological inhibition of LPA1 led to a substantial reduction in tumor size and blocked metastases [56, 57]. Recent studies have revealed that an LPA1 antagonist effectively suppressed cell survival, migration, and invasion in the TNBC cell line, without triggering apoptosis in the TNBC cells. Additionally, it exhibited no cytotoxic effects, highlighting the promising potential of LPA1 as a migrastatic target for TNBC [58].

For molecular generation, we selected low loss weights for property prediction (auxiliary) modules and latent $l_2$ penalty, as they serve to regularize the latent space and are not deemed as significant as reconstructive loss or contrastive learning. Five executions for each molecular generation model were performed, with the random seeds displayed in Table 3. Since we included both the base ReLSO and a contrastive learning ReLSO variant, this consists of 15 total executions. For our molecular generation experiments we employ the SELFIES notation [17], as preliminary experiments show poor molecular validity for downstream optimization with SMILES.

To train our latent Transformer molecular generation models, we used the dataset with 4 million unique canonicalized molecules from ZINC-250K [44], ChEMBL [45], and MOSES [46] datasets. We applied two filters for pre-processing: (1) exclude molecules with a tokenization length greater than 198, and (2) retain only molecules that have at least two unique augmentations within 10 attempts. After pre-processing, three molecular properties were calculated using RDKit [40] for property prediction and regularization of the latent space: SAS, logP, and QED [59]. QED is a weighted sum of properties that evaluate the drug-likeness of a molecule, scaled between 0 and 1, and logP is a measure of lipophilicity. The pre-processed dataset was afterwards divided into a 70% training, 10% validation, and 20% test split. During experimentation, we used cross entropy loss for reconstruction, mean squared error loss for property prediction, which are all regression tasks, and NT-Xent loss for contrastive learning. We opted to train our models until validation loss increases after four consecutive epochs, and perform a validation epoch every 20% of training epoch steps.

Table 4 outlines the parameters used for the six metaheuristic algorithms employed during our drug design experiments. All metaheuristics use simulated binary crossover (SBX) [60] and polynomial mutation [61], with the evolutionary algorithms using binary tournament selection. As well, crossover probabilities $p_c = 1$, mutation probabilities $p_m = 1/D$, where *D* is the number of decision variables, and both operator distribution indices $n_c = n_m = 20$. We note that A-NSGA-III is not included in Table 4 as all its hyperparameter values are described.

During our drug design experiments, we pre-trained and fine-tuned the MTL-BERT model [42] for ADMET prediction using the hyperparameters (see Table 5) expressed in their medium set. To construct the model, we used the same 4 million pre-training dataset as the molecular generation models, however used a 80-20 train-test split and SMILES notation instead of SELFIES. In addition, we did not perform SMILES enumeration for training MTL-BERT. During fine-tuning of MTL-BERT using data (see Table 6) from [47], we used fivefold cross validation, and selected the fold with the best performance on downstream tasks for our drug design system. Following a similar strategy to molecular generation, we terminated training after two test epochs with a consecutive increase in loss value. During pre-training, we performed a test epoch every 5000 training steps, while during fine-tuning, a test epoch was executed after each training epoch.

**Table 5:** MTL-BERT hyperparameters

Name	Value
Pre-train learning rate	1e−4
Fine-tune learning rate k	5e−5
Embedding dimension	256
Transformer layers	8
Self-attention heads	8
Feedforward dimension	1024
Dropout	0.1
Batch size	64
Random seed	42

**Table 6:** Summary of datasets for MTL-BERT fine-tuning

ADMET	Dataset name	Size	Type
Absorption	Caco-2	824	Regression
Absorption	PAMPA Permeability	1725+/286−	Classification
Absorption	HIA	493+/59−	Classification
Absorption	Pgp inhibition	631+/547−	Classification
Absorption	Bioavailability	478+/127−	Classification
Absorption	Lipophilicity	4189	Regression
Absorption	Solubility	9757	Regression
Absorption	FreeSolv	642	Regression
Distribution	BBB	1521+/411−	Classification
Distribution	PPBR	1600	Regression
Distribution	VDss	1036	Regression
Metabolism	CYP 2C19	5783+/6625−	Classification
Metabolism	CYP 2D6	2491+/10379−	Classification
Metabolism	CYP 3A4	5036+/7055−	Classification
Metabolism	CYP 1A2	5822+/6502−	Classification
Metabolism	CYP 2C9	4012+/7817−	Classification
Metabolism	CYP2C9 substrate	140+/493−	Classification
Metabolism	CYP2D6 substrate	189+/442−	Classification
Metabolism	CYP3A4 substrate	330+/302−	Classification
Excretion	Half life	591	Regression
Excretion	Hepatocyte clearance	1196	Regression
Excretion	Microsome clearance	1099	Regression
Toxicity	LD50	7362	Regression
Toxicity	hERG	443+/195−	Classification
Toxicity	AMES	3961+/3289−	Classification
Toxicity	DILI	228+/232−	Classification
Toxicity	Skin reaction	274+/130−	Classification
Toxicity	Carcinogens	51+/188−	Classification
Toxicity	ClinTox	100+/1232−	Classification

### Comparing transformers for latent space generation

To evaluate the performance of the molecular generation models, we used loss values among all the joint training tasks as metrics. For molecular reconstruction, we also included accuracy of token prediction. Furthermore, we provided visualizations of the latent space on the validation and test set. To achieve this, we applied principal component analysis (PCA) to reduce dimensionality to 50, and t-distributed stochastic neighbor embedding (t-SNE) [62] for reduction to two dimensions, as was done in [63]. Similarly, Uniform Manifold Approximation and Projection (UMAP) [64] was applied for reduction to three dimensions after PCA. This allows us to view the organization of the latent space by each of the molecular properties predicted in the auxiliary networks.

**Fig. 4:**
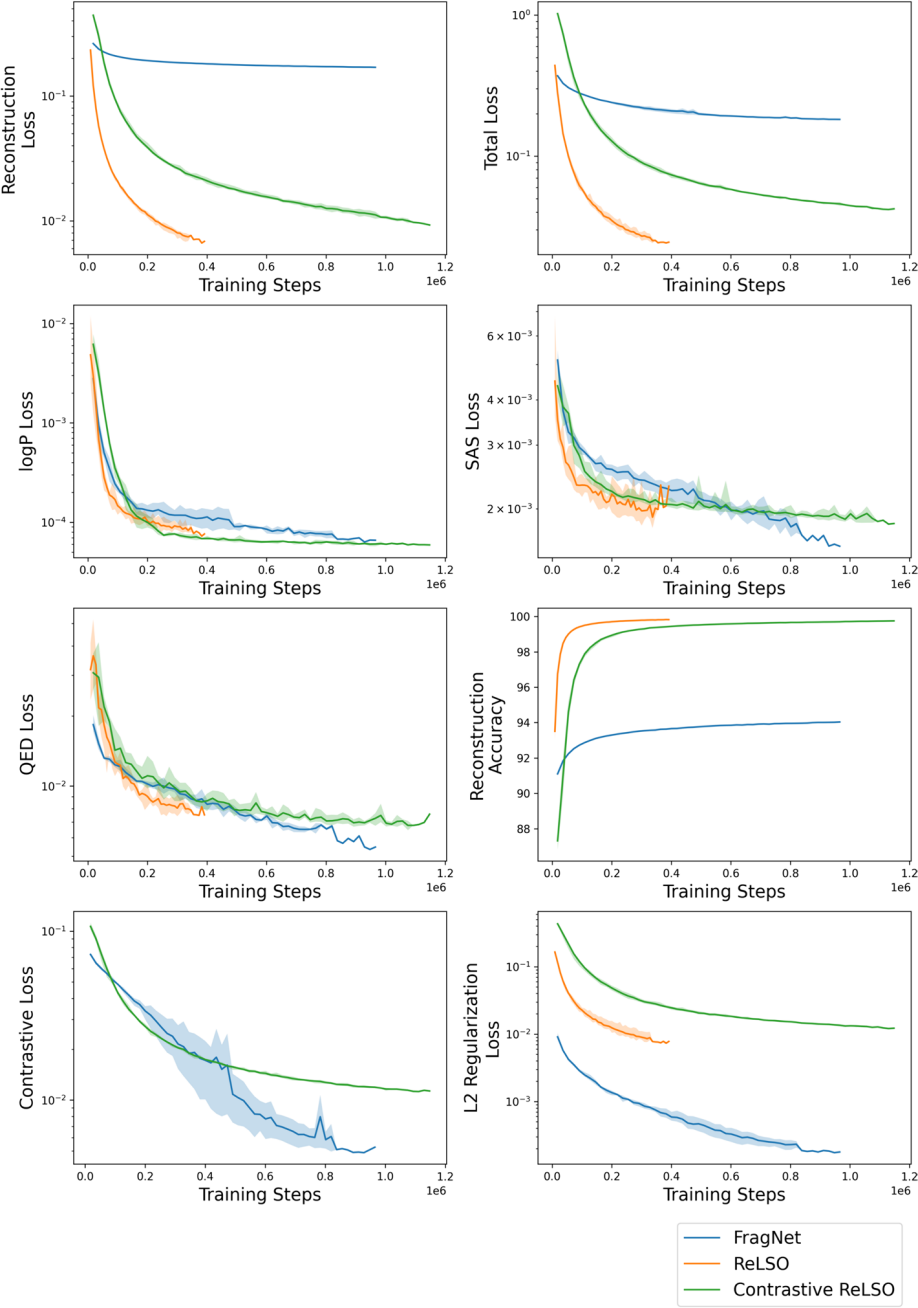
Comparison between ReLSO, contrastive ReLSO, and FragNet by validation set performance markers

**Table 7:** Comparison between ReLSO, contrastive ReLSO, and FragNet by mean test set performance markers

Metric	ReLSO	Contrastive ReLSO	FragNet
logP prediction loss	8.88e−5	**6e**−**5**	7.81e−05
SAS prediction loss	2.03e−3	1.92e−3	**1.79e**−**03**
QED prediction loss	7.9e−3	7.43e−3	**6.50e**−**03**
Contrastive loss	–	0.011582	**0.0101**
Reconstruction loss	**7.34e**−**3**	0.0106	0.173
Reconstruction accuracy	**99.81**	99.72	93.9
Latent l2 loss	8.33e−03	0.0131	**2.74e**−**04**
Total test loss	**0.0257**	0.0447	0.195

Best value in each metric is highlighted in bold

Table 7 presents the mean performance of each molecular generation model on the test set. Property prediction losses are low, with FragNet achieving the lowest values for SAS and QED, and Contrastive ReLSO for logP. As well, both ReLSO models surpass FragNet in terms of reconstruction loss and accuracy, a crucial metric shared by all three models. Figure 4 illustrates the mean validation set performance during training, and shows similar outcomes between FragNet and ReLSO models. The architectural differences, especially in the decoder and projection modules, likely account for the differing molecular reconstruction abilities. ReLSO employs a convolutional decoder directly from the latent space, whereas FragNet uses an unprojection module, followed by a Transformer decoder. Moreover, ReLSO applies pooling to the output of the Transformer encoder before projecting to the latent space, while FragNet projects directly after the encoder. These modifications in the ReLSO model likely enhance its reconstruction capability. On latent $l_2$ regularization loss, another metric shared by all experiments, FragNet performs the best. Regarding the contrastive loss, which is not applicable to the base ReLSO as it does not consider the task, FragNet slightly outperforms the contrastive ReLSO model. Upon comparison of ReLSO and its contrastive variant, we view that contrastive learning slightly reduces reconstruction capabilities, with an increase in loss and decrease in accuracy, likely in favour of organization within the latent space. Since reconstruction is the most crucial task for a latent Transformer model, the ReLSO models offer better overall performance. Additionally, ReLSO performs well on the contrastive learning objective, as evidenced by the values in Table 7.

Figure 4, which illustrates the mean and standard deviation by a shaded colour, indicates a higher variability among the FragNet experiments, particularly in the contrastive loss. It is important to mention that the experiments are terminated when overfitting occurs on the validation set, rather than after a fixed number of epochs. Therefore, the shaded regions may vanish or shrink as the number of training steps increases. According to the plots, FragNet achieves the lowest contrastive, SAS, QED, and $l_2$ regularization losses, but it exhibits poor reconstruction performance, as shown in Table 7. The reconstruction loss curve for FragNet reaches a plateau much earlier than the ReLSO models, possibly due to a local minima. The original FragNet study used a much smaller training dataset and trained for only one epoch, which differs from the current experiments. Even with the same learning rate as the original study, the FragNet experiments in this work have a significantly higher reconstruction loss. However, the contrastive losses are comparable between this study and the original. Similar to the test set, we see that the ReLSO models outperform FragNet in the crucial task for molecular generation, which is reconstruction, while being competitive in contrastive learning.

**Fig. 5:**
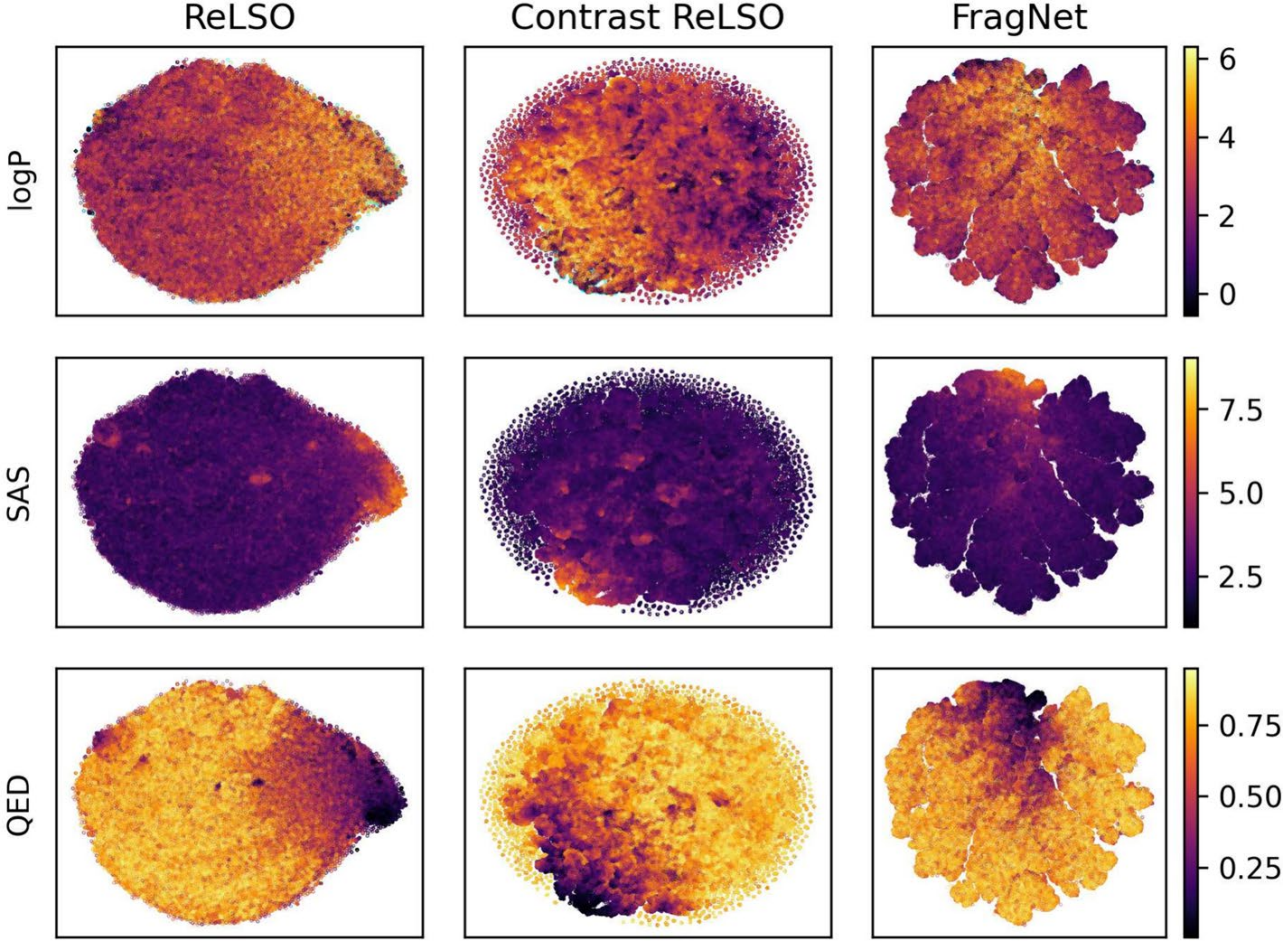
Latent space visualization of ReLSO, contrastive ReLSO, and FragNet on validation and test data in 2D space using t-SNE

**Fig. 6:**
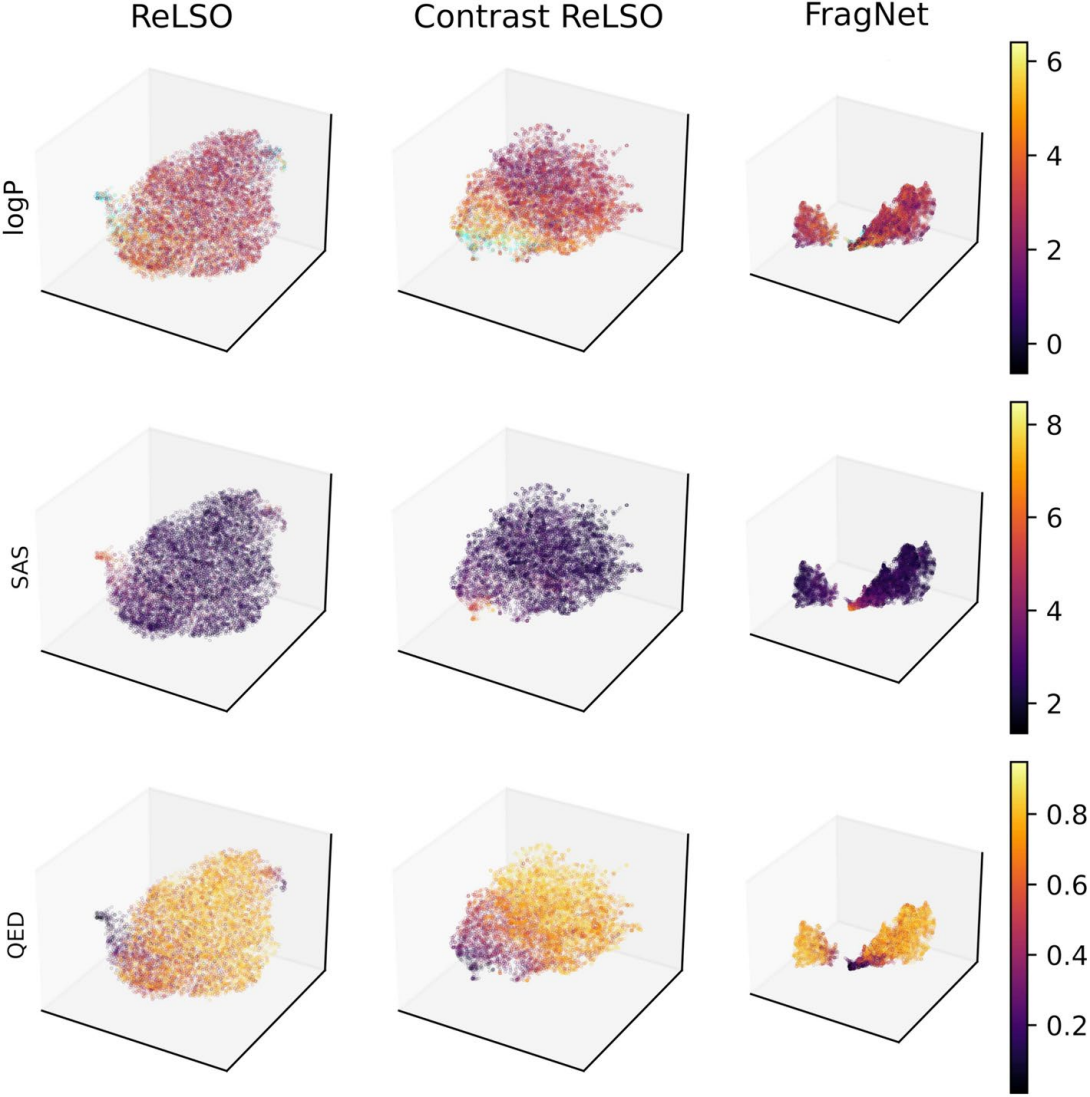
Latent space visualization of ReLSO, contrastive ReLSO, and FragNet on validation and test data in 3D space using UMAP

Figures 5 and 6 illustrate latent space organization by molecular property values on the validation and test sets using t-SNE [62] and UMAP [64], respectively. From both, we observe clear trends among all three models for SAS and QED organization. A negative correlation exists between the QED and SAS values in similar areas of the latent space, which demonstrates a tendency where molecules with low drug-likeness have probable difficulties in synthesis. LogP is not as well organized as QED and SAS. There are regions of distinct high and low logP values from all models, but they are not as efficiently organized as QED and SAS. Contrastive ReLSO has the best attempt at clustering low logP values, compared to ReLSO and FragNet, with unambiguous areas of low and high values. In addition, we use cyan to denote the logP values that are considered outliers on a boxplot analysis. Outlier points are mainly located near high logP and low QED regions. This is reasonable, since highly lipophilic molecules have been found to be poor drug candidates [65], and should be related to a lower drug-like score. From the 3D visualization in Fig. 6, FragNet shows two clusters, indicating that the latent variable distribution may have multiple components, which may cause extra difficulty for search algorithms in the latent space. In summary, all three models have a good organization of the latent space with respect to property values. ReLSO and FragNet have smoother transitions over a larger area, while Contrastive ReLSO has sharper boundaries that separate the patterns. Furthermore, FragNet shows disconnected components.

### Comparing computational intelligence methods for molecular optimization

**Fig. 7:**
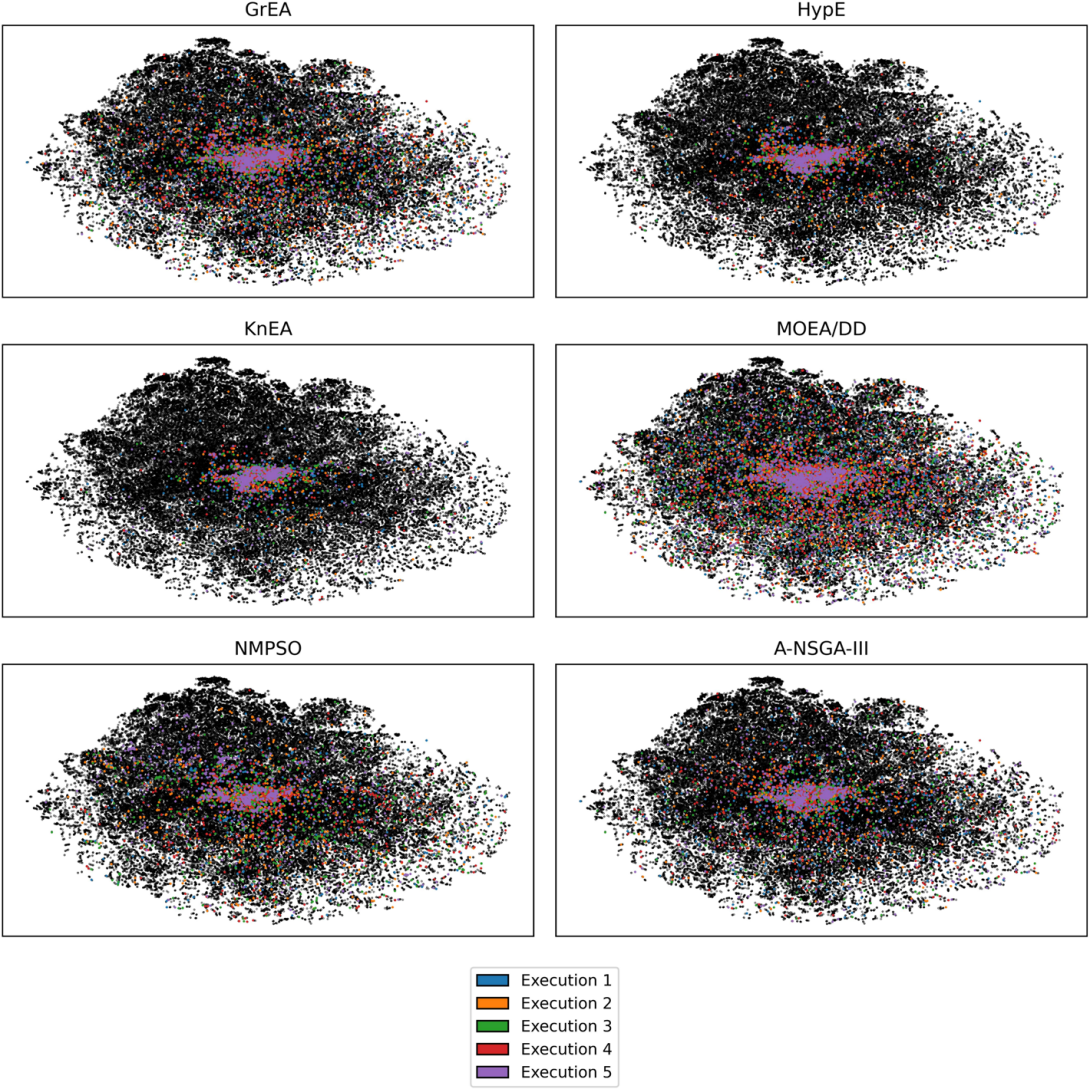
Latent space coverage of final populations generated by metaheuristic algorithms, categorized by execution. Black points represent latent representations of test samples. These 2D maps were generated using t-SNE

To assess the quality of solutions obtained by the metaheuristic algorithms, we employ latent space coverage visualizations, generational distance (GD), inverted generational distance (IGD), uniqueness, novelty, Wasserstein distance, and density plots of objective values. Uniqueness is defined as the portion of unique molecules among a population of generated molecules. Novelty gives the portion of novel (not in training set) molecules among a population of generated molecules. GD and IGD measure how well the approximated front matches the true Pareto front, where lower values are better. Since the real Pareto front is unknown, we take the non-dominated solutions among all metaheuristic experimentation, and use this as an approximation. Uniqueness and novelty measure the amount of unique molecules in each population and the proportion of molecules not in the molecular generation dataset, respectively. For both of these measures, higher is typically better. Wasserstein distance is a metric that captures the distance between probability distributions, which we display alongside density plots that illustrate the distributions of objective values.

An examination of Fig. 7 reveals the latent space coverage across the final populations in five separate executions, corresponding to each metaheuristic algorithm. First, the illustration serves to highlight the impact of varying initial populations on the performance of each algorithm. For each algorithm, all results of the five runs concentrates at the same area of the chemical space with moderate variations. Second, among the six algorithms, different levels of coverage can be observed. It is evident that MOEA/DD, A-NSGA-III, NMPSO, and GrEA exhibit larger latent space coverage, whereas HypE and KnEA demonstrate concentrated coverage at the common area. This disparity reflects that certain metaheuristic algorithms have greater exploration ability in the search space, while others focus more on exploitation. Remarkably, MOEA/DD stands out with the most extensive latent space coverage, as indicated by the numerous points dispersed throughout the search space. Additionally, there is a notable concentration of points around the central region for all algorithms utilized, suggesting the need for further trials with alternative metaheuristic algorithms to identify those capably of more effectively probing the extremeties of the search space.

**Fig. 8:**
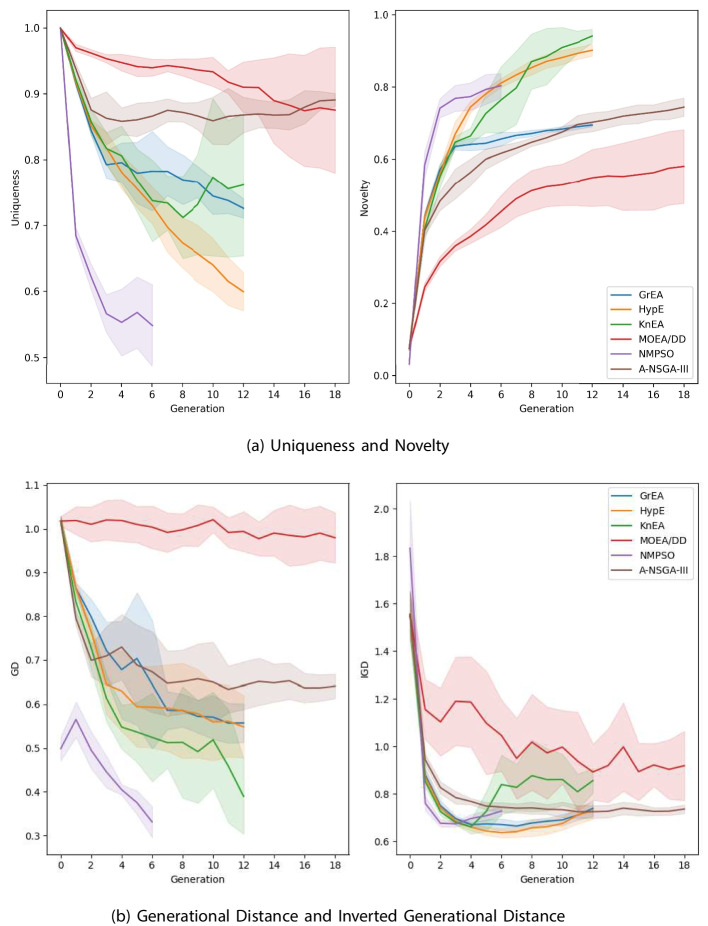
Metaheuristic performance comparison on **a** uniqueness and novelty of molecules and **b** generational distance and inverted generational distance. Solid curves represent the mean over five runs, and shaded regions express the standard deviation

Figure 8 illustrates the generational performance of each metaheuristic over (a) average uniqueness and novelty, and (b) average GD and IGD metrics. We observe that uniqueness of the solutions decrease for all algorithms during the optimization process, indicating that similar molecules are generated and retained throughout generations. However, some algorithms, such as A-NSGA-III and KnEA, show a slight increase in uniqueness after an initial decline, maintaining a high level of diversity among the solutions. As anticipated, the novelty of solutions increase from the initial population, which consists of randomly sampled molecules from the molecular generation dataset that are not likely to be novel, suggesting that the algorithms are exploring new regions of the search space. After the optimization process starts, populations are quickly filled with higher amounts of unseen molecules, with NMPSO, KnEA, and HypE, those with the most novel, nearing or surpassing rates of 80%. Furthermore, in Fig. 8, shaded regions express the standard deviation of generational performance. We can see that MOEA/DD and KnEA show relatively larger variations, while the other four algorithms are more stable.

Regarding GD and IGD, all algorithms on average improve from their initial population, but MOEA/DD exhibits a notably poor approximation of the Pareto front throughout all generations, as evidenced by its high GD values. This observation is less extreme in the IGD metric, however MOEA/DD still performs the worst among remaining algorithms, even with its high coverage of the search space. The remaining algorithms have similar Pareto-approximation performance measures, but NMPSO and KnEA clearly outperform the others on GD, followed by HypE, GrEA, and A-NSGA-III. For IGD, the final result is less clear. It is noted that for all algorithms, Pareto approximation improves significantly within the first two generations, marked by a slight deterioration afterwards. This coincides with the generation where the uniqueness of the solutions declines. It is possible that a change in parameters, along with additional functional evaluations, could enhance exploration of the search space before exploiting the optima. Nevertheless, NMPSO, GrEA, and HypE perform best on the IGD metric, ending with similar values.

**Table 8:** Average runtime across five metaheuristic experiments

Algorithm	Average runtime (h)
GrEA	5.53
HypE	5.71
KnEA	5.34
MOEA/DD	4.84
NMPSO	6.54
A-NSGA-III	4.94

The experiments were conducted using a computer with AMD Ryzen 7 7700x CPU, RTX 4090 GPU, and 32 GB DDR5 RAM

When comparing convergence of the algorithms, both GD and IGD provide conflicting information. For NMPSO and KnEA, there is room for additional improvement on GD, however this is not the case with IGD. The other algorithms, such as A-NSGA-III, GrEA, and HypE, appear close to converging on GD, but degrade on IGD. It is likely that increasing the functional evaluations would improve the approximated Pareto front, however due to the long computational time required for these experiments, such as expressed in Table 8, this is left for future work.

**Fig. 9:**
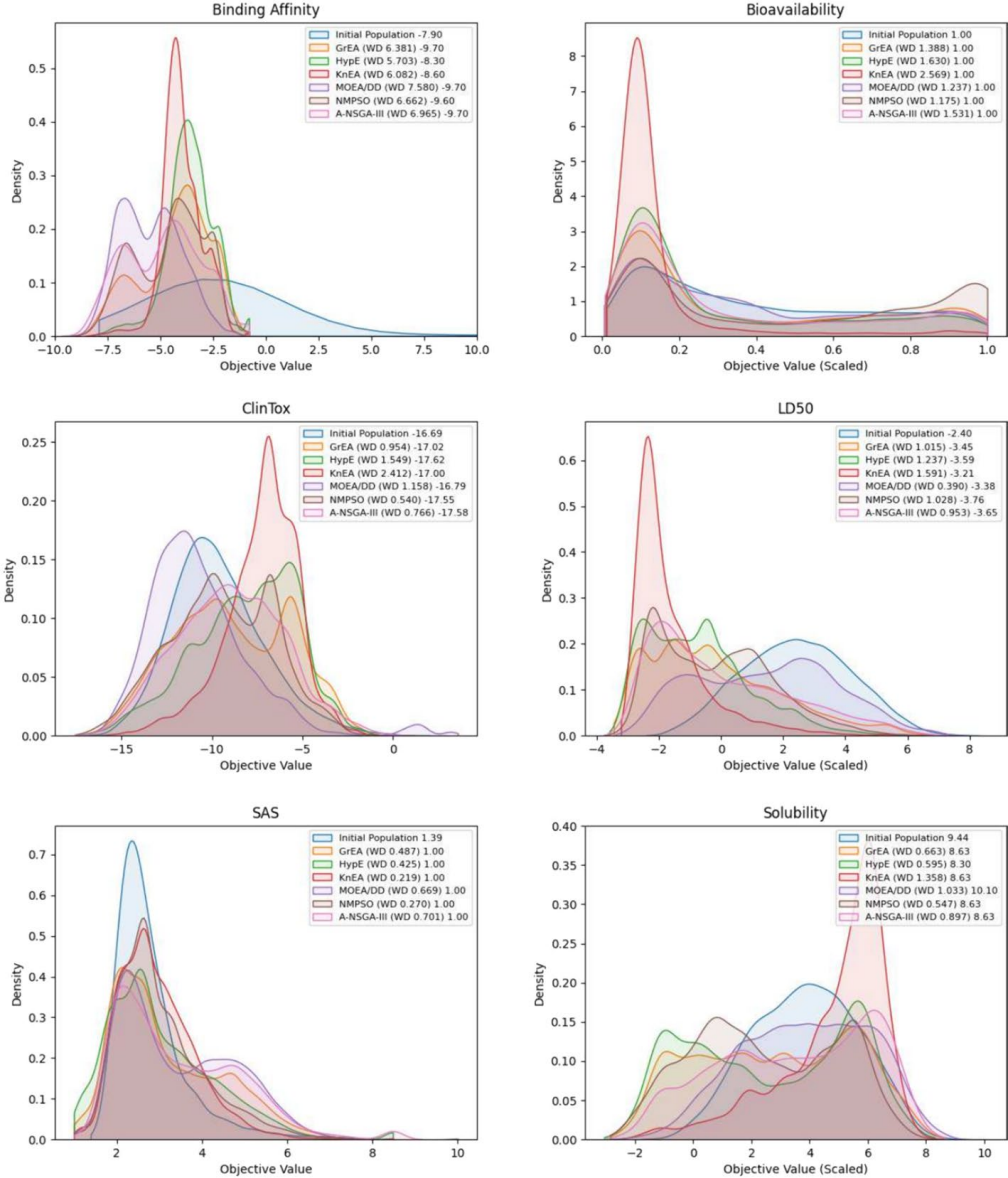
Objective distributions with 1-Wasserstein distances between initial and final populations, and best objective values

Figure 9 illustrates kernel density plots, best value of the final and initial populations, and 1-Wasserstein distance values between the combined final population distributions and initial distribution, per each objective. For a description of which objectives are maximization or minimization, we refer the reader to "Many-objective drug design from the latent space" section. For all objectives except solubility, the final populations contain solutions that are equal to or better than the initial population. On the solubility objective, MOEA/DD is the only metaheuristic that obtains a value higher than the initial best. Moreover, the Wasserstein distance between the initial and final populations increases significantly, indicating that the metaheuristics have explored various regions of the search space to obtain a large spread of solutions. This is likely due to the use of a Pareto-dominance relation to guide the search, and return a diverse set of trade-off solutions. It is noteworthy that MOEA/DD, despite having the worst performance in terms of GD and IGD Pareto approximation metrics, is able to obtain a comparable best value for many of the drug design objectives, such as bioavailability, SAS, binding affinity, LD50, and solubility.

Binding affinity, a critical measure that incorporates ligand-target information, is substantially improved between the initial and final populations, with the best values achieved by A-NSGA-III, GrEA, MOEA/DD, and NMPSO. Interestingly, on the acute toxicity LD50 objective, many of the metaheuristics have distributions that are concentrated in the negative values, indicating low toxicity. This contrasts with the results on the ClinTox objective, where the resulting metaheuristic distributions are still negative, but concentrated in higher values than the initial population. Due to the use of the Pareto-dominance relation, the objective values of the final populations tend to have a larger spread than the initial population, which is also reflected with higher Wasserstein distance values. It is observed that, except for SAS, metaheuristics are able to find more solutions with poor objective values than with adequate ones. For instance, in bioavailability, where higher values are preferable, many algorithms have values concentrated closer to zero. This reflects the complexity of drug design, where the chemical space is large, and although the metaheuristic algorithms find novel molecules, many of them have poor ADMET properties. Among all algorithms, which employ different approaches to many-objective optimization, NMPSO and A-NSGA-III consistently find solutions with the best values for each individual objective.

### Case study

**Fig. 10:**
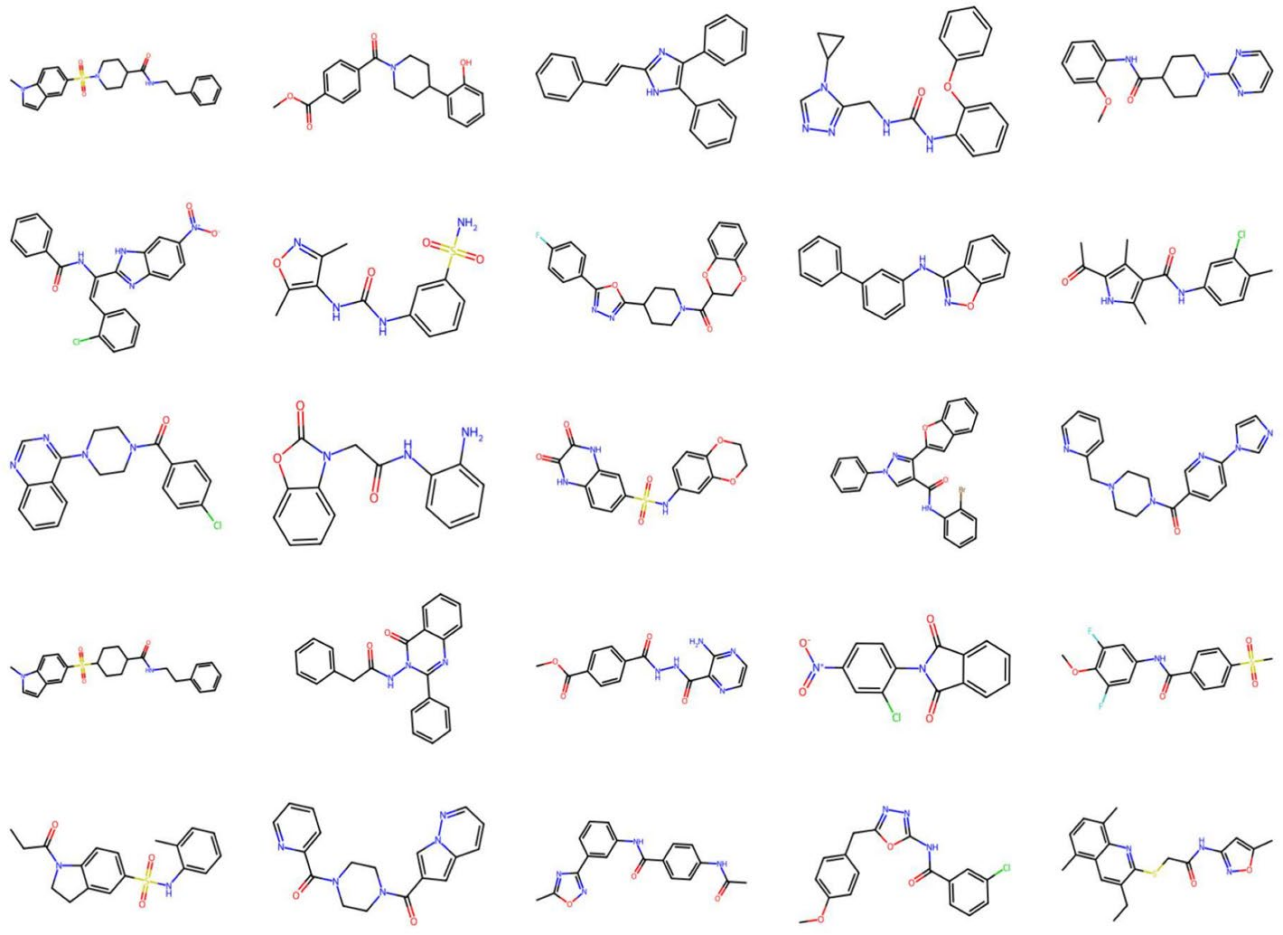
2D graph visualization of top 25 high-quality filtered molecules from final population of metaheuristics after application of normalized sum of ranks

**Fig. 11:**
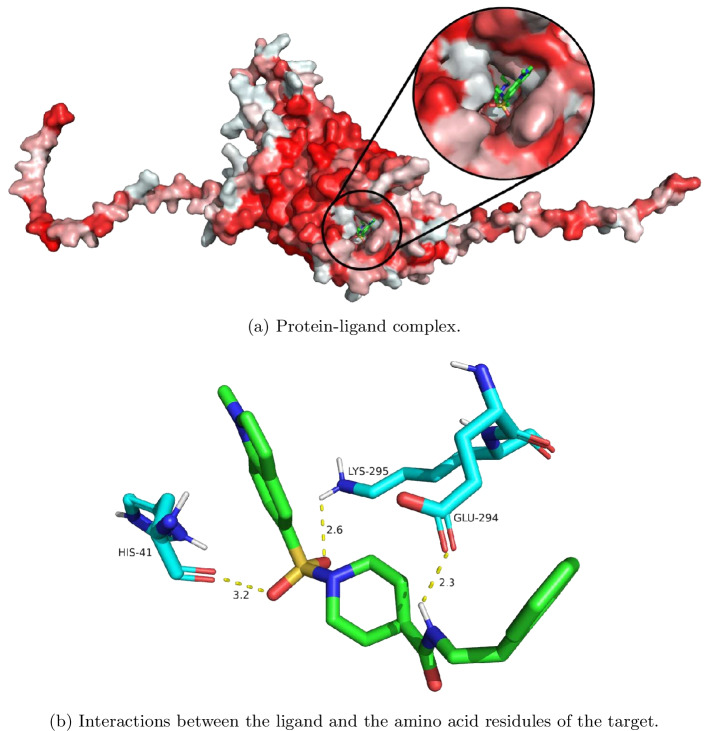
Docking visualization of top filtered ligand (Cn1ccc2cc(S(=O)(=O)N3CCC(C(=O)NCCc4ccccc4)CC3)ccc21) in complex with the LPA1 protein binding pocket. The 2D structure of the ligand is displayed as the first graph in Fig. 10. **a** Illustrates the molecular surface of LPA1 protein coloured by Einsenberg’s scale [67], where red indicates higher hydrophobicity, along with the binding pose and location of candidate ligand, while **b** shows molecular interactions between the molecule and amino acid residues within the LPA1 pocket

Upon obtaining the final populations, we apply a filtering process to remove molecules with poor lipophilicity, as measured by logP, poor SAS score, and poor binding affinity. For this, we employ the Ghose filter ($-\,0.4 \le {\text {logP}} \le 5.6$) [66], binding affinity filter ($\le$ $-$ 7.1), and SAS filter ($\le 3$). The threshold $-$ 7.1 for LPA1 was obtained by the docking scores of known LPA1 inhibitors and then taking the largest docking score among them. Using the binding filter for a virtual screening on the ZINC lead-like data, we found that 25% of molecules may bind to the LPA1 protein. After application of these filters, 1718 molecules remain, with 20.8%, 2.4%, 3.0%, 44.4%, 12.2%, and 17.1% from GrEA, HypE, KnEA, MOEA/DD, NMPSO, and A-NSGA-III, respectively. Interestingly, a large proportion of the filtered molecules come from MOEA/DD. As previously discussed, MOEA/DD did not obtain a good Pareto-front approximation as measured by GD and IGD, however had relatively strong performance on each individual drug design objective and high latent space coverage within its final populations. After filtering molecules, they are organized using a normalized sum of ranks scheme on their corresponding objective vectors. The unique, 25 highest performing molecules are displayed in Fig. 10. As well, we include an image of the best performing molecule (first molecule from Fig. 10), obtained from A-NSGA-III, in complex with the LPA1 protein in Fig. 11, along with highlighting interactions with protein residues.

## Conclusion

In this paper, we propose a comprehensive system for drug design, based on two experimental studies. The first study compares three latent Transformer models for molecular generation: a contrastive learning and a non-contrastive learning variants of ReLSO, and FragNet. Two of these models, contrastive ReLSO and FragNet, exploit the non-uniqueness property of SMILES representations and employ contrastive learning as a latent space regularizer. Using non-contrastive ReLSO as a baseline model, we examine the impact of contrastive learning on molecular representation learning. The second study compares several many-objective metaheuristic algorithms for drug design. We integrate an ADMET prediction model, a molecular generation model, a molecular docking algorithm, and a metaheuristic algorithm to form a complete system for drug design. Our experimental results show that ReLSO outperforms FragNet as a molecular generation model, and that MOEA/DD shows promising results as a many-objective metaheuristic algorithm for drug design. MOEA/DD achieves among the highest objective values and the highest percentage of molecules that pass our three filters, despite not obtaining good Pareto-front approximations. We suggest that future work should conduct a comparative study other recent molecular generation models, and analyze the scalability of drug design objectives and metaheuristics. We also hypothesize that the performance of our system can be enhanced by adopting the DEL framework and evolutionary dynamic optimization algorithms.

## Data Availability

The molecular data used in this research is a combined set of the MOSES, ChEMBL, and ZINC-250K databases (accessible via https://tdcommons.ai). The structure of LPA1 was obtained from the AlphaFold Protein Structure Database (AlphaFold Entry: Q92633). The implementation of this research can be found at https://github.com/Pixelatory/ManyObjectiveDrugDesign. No datasets were generated or analysed during the current study.

## Abbreviations

A-NSGA-III: Adaptive non-dominated sorting genetic algorithm III
ADMET: Absorption, distribution, metabolism, excretion, and toxicity
AI: Artificial intelligence
BERT: Bidirectional encoder representations from transformers
DEL: Deep evolutionary learning
FragNet: A contrastive learning-based transformer model for clustering, interpreting, visualizing, and navigating chemical space
GD: Generational distance
GrEA: Grid-based evolutionary algorithm
HypE: Hypervolume estimation
IGD: Inverted generational distance
KnEA: Knee point-driven evolutionary algorithm
logP: Log octanol-water partition coefficient
LPA1: Lysophosphatidic acid receptor 1
MOEA/DD: Multi-objective evolutionary algorithm based on dominance and decomposition
MSO: Molecule swarm optimization
MTL-BERT: Multi-task learning BERT
NMPSO: Novel multi-objective particle swarm optimizer
NT-Xent: Normalized temperature-scaled cross entropy
PCA: Principal component analysis
PlatEMO: Evolutionary multi-objective optimization platform
PSO: Particle swarm optimization
QED: Quantitative estimate of drug-likeness
ReLSO: Regularized latent space optimization
RNN: Recurrent neural network
SAS: Synthetic accessibility score
SELFIES: SELF-referencing embedded string
SMILES: Simplified molecular-input line-entry system
t-SNE: T-distributed stochastic neighbor embedding
UMAP: Uniform manifold approximation and projection
